# Hyperinsulinaemic hypoglycaemia in non-anaesthetized Göttingen minipigs induces a counter-regulatory endocrine response and electrocardiographic changes

**DOI:** 10.1038/s41598-021-84758-w

**Published:** 2021-03-16

**Authors:** Mille K. Lyhne, Andreas Vegge, Gro Klitgaard Povlsen, Rita Slaaby, Jonas Kildegaard, Ulrik Pedersen-Bjergaard, Lisbeth H. Olsen

**Affiliations:** 1 Department of Veterinary and Animal Sciences, University of Copenhagen, Copenhagen, Denmark; 2 Global Drug Discovery, Novo Nordisk A/S, Måløv, Denmark; 3 Department of Endocrinology and Nephrology, Nordsjællands Hospital Hillerød, Hillerød, Denmark

**Keywords:** Endocrine system and metabolic diseases, Cardiovascular biology, Diabetes complications

## Abstract

The potentially fatal cardiovascular effects of hypoglycaemia are not well understood and large animal models of the counter-regulatory responses and cardiovascular consequences of insulin-induced hypoglycaemia are needed to understand the mechanisms in humans. The aim of this study was to develop a human-like minipig model of hypoglycaemia including healthy and diabetic pigs to investigate endocrine, electrocardiographic and platelet effects. Hypoglycaemia was induced using a hyperinsulinaemic, hypoglycaemic clamp and an insulin bolus protocol. Plasma glucose, glucagon, C-peptide, insulin, epinephrine and platelet aggregation responses were measured before, during and after hypoglycaemia. Continuous electrocardiographic recordings were obtained. Hypoglycaemia at a plasma glucose concentration of 0.8–1.0 mM in the clamp induced 25-fold increase in epinephrine and sixfold and threefold increase in glucagon for healthy and diabetic pigs, respectively. The hypoglycaemic clamp induced QTc-interval prolongation and increase in cardiac arrhythmias. In the bolus approach, the non-diabetic group reached plasma glucose target of 1.5 mM and QTc-interval was prolonged after insulin injection, but before glucose nadir. The diabetic group did not reach hypoglycaemic target, but still demonstrated QTc-interval prolongation. These results demonstrate effects of hyperinsulinaemic hypoglycaemia closely resembling human physiology, indicating the minipig as a translational animal model of counter-regulatory endocrine and myocardial effects of hypoglycaemia.

## Introduction

Hypoglycaemia is a common adverse effect of insulin treatment in type 1 and type 2 diabetes with serious clinical implications for the patient^1^. People with diabetes also have a higher risk of cardiovascular disease than the general population, as well as micro- and macroangiopathies, whose pathogeneses has been linked to increased platelet activation^2, 3^. Large-scale clinical studies have demonstrated an increased risk of overall mortality and cardiovascular death in people with diabetes experiencing as little as a single severe hypoglycaemic episode, even days and months after the event^1, 4, 5^, where severe hypoglycaemia was defined as blood glucose below 2.8 mM with the person needing third-party assistance to return to normal blood glucose levels^1^. The majority of data linking diabetes to cardiovascular risk have been generated in type 2 diabetes patients, potentially due to large cardiovascular outcome trials in this populations, however type 1 diabetes patients also have an increased cardiovascular risk compared to the non-diabetic population, including higher risk of cardiovascular death^6^. The mechanisms underlying this association remains poorly understood, but is thought to include an acute proarrhythmic effect of hypoglycaemia and more long-lasting effects promoting inflammation, coagulation and atherosclerosis^7^. The acute cardiovascular effects of hypoglycaemia include increased cardiac workload driven by the counter-regulatory epinephrine response and QTc-interval prolongation in healthy and diabetic humans^8–10^. Furthermore, both experimental and spontaneous hypoglycaemia in humans induces cardiac arrhythmic ventricular and supraventricular ectopic electric activity^11, 12^. The dead-in-bed syndrome in young type 1 diabetes patients has been linked to hypoglycaemia-induced, fatal cardiac arrhythmias^11, 13^. However, there are no data to definitely prove causality. In rodents, severe hypoglycaemia induces fatal arrhythmias seemingly mediated by the para- and sympathetic nervous system^14, 15^, but the effects of hypoglycaemia has not been well investigated in a large animal model with more human-like anatomy and physiology. The Göttingen minipig is widely used in preclinical research^16^, and their cardiac anatomy and physiology closely resembles that of humans, with cardiac conduction systems and heart size being very similar^17, 18^. In addition, porcine models appear to resemble the human cardiovascular system better than the rodent models since resting heart rate in minipigs is 85–99 beats per minute compared to the rat’s mean heart rate of 350 beats per minute. Furthermore QTc-interval length in minipigs (363–383 ms)^17^ is more similar to humans (360–460 ms (female))^19^ than rats (127–143 ms)^14^. While the minipig is frequently used to test new pharmacological agents for diabetes treatment, only few studies have focused on modelling diabetic comorbidities and no studies have been conducted investigating the effects of insulin-induced hypoglycaemia. A translational large animal model of hyperinsulinaemic hypoglycaemia, to investigate pathological consequences of hypoglycaemia—severe hypoglycaemia in particular—and test possible interventions, has yet to be developed.

The aim of this study was to develop a human-like minipig model of hypoglycaemia in non-anaesthetized healthy and streptozotocin-induced diabetic Göttingen minipigs using two different protocols of insulin-induced hypoglycaemia, hypothesizing that hypoglycaemia induces a human-like counter-regulatory hormonal response, cardiac arrhythmias, electrocardiographic T-wave, QT interval and ST-segment changes, heart rate variability and increased platelet aggregation response, as has previously been shown in human clinical studies^9, 20–22^.

## Materials and methods

### Animals

All animal studies have been approved by the Danish Animal Experiment Inspectorate and conducted in accordance with rules and regulations set forth by the inspectorate. Fourteen intact female, adult, lean Göttingen minipigs (Ellegaard Göttingen Minipigs A/S, Dalmose, Denmark) were included in this study. Prior to enrollment, pigs were surgically implanted with two permanent central venous catheters (Cook C-TPNS-6.5-90-REDO, William Cook Europe ApS, Bjæverskov, Denmark) and one permanent auricular vena jugularis catheter. Using the Seldinger technique, central catheters were advanced trough the vena cava cranialis, through the right atrium and into the vena cava caudalis (modified from Larsen et al. 2002^23^).

Eight pigs were kept as healthy controls (CON) and six were made hyperglycemic (DIA) three months prior to this study by once-daily i.v. injection of 50 mg/kg streptozotocin (Sigma Aldrich Denmark A/S, cat. no. S0130-5G) for three consecutive days (modified from Schumacher et al*.* 2019^24^). The pigs in the DIA group had been used for other pharmacological studies prior to this study, with a washout period of one month between studies. In DIA, hyperglycaemia was managed by once-daily measurement of plasma glucose and s.c. injection of insulin glargine (Lantus, Sanofi S.A., Paris, France) in conjunction to feeding, based on individual plasma glucose curves, to maintain target fasting plasma glucose of 9–12 mM. Pigs were fed 460 g of feed once-daily (SDS minipig, Special Diets Service, Essex, UK) and were single-housed with wood chip and straw bedding, possibility of snout-contact and free access to water.

### Hypoglycaemia protocols

Hypoglycaemia was induced using two different protocols: hyperinsulinaemic, hypoglycaemic clamp (CLAMP) and a single bolus insulin exposure (BOLUS) with at least 14 days wash-out period between each protocol. Pigs were fasted for 24 h prior to study. The day prior to study DIA received a low dose of 15 U insulin glargine to control hyperglycaemia while ensuring that none of the pigs would be hypoglycaemic at the day of study.

During CLAMP, pigs were given a continuous i.v. infusion of human insulin at 16 pmol/kg/min in the auricular catheter. The dosage was determined from the linear phase of a dose–response curve of insulin infusion rate-glucose infusion rate generated in a pilot study of both healthy and diabetic minipigs (data not shown). Plasma glucose (PG) levels were controlled by individually adjusting i.v. glucose infusion rate (GIR) in one of the central vena cava catheters, based on PG measured every five to fifteen minutes during CLAMP. Glucose levels were first clamped to a minipig normoglycaemic target (3.5 mM) for three hours. To induce acute hypoglycaemia, GIR stopped, and gradually adjusted to keep PG at target 0.8–1.0 mM for two hours. The study was finalized by two hours of normoglycaemia, where after all infusions were turned off.

For the BOLUS protocol, a single bolus of human insulin was dosed i.v. and PG was monitored. Insulin dose targeted PG < 1.5 mM was determined in a pilot study in healthy pigs (data not shown). Healthy pigs received 0.4 nmol/kg human insulin and diabetic pigs 0.6 nmol/kg, due to expected lower insulin sensitivity.

**Figure 1:**
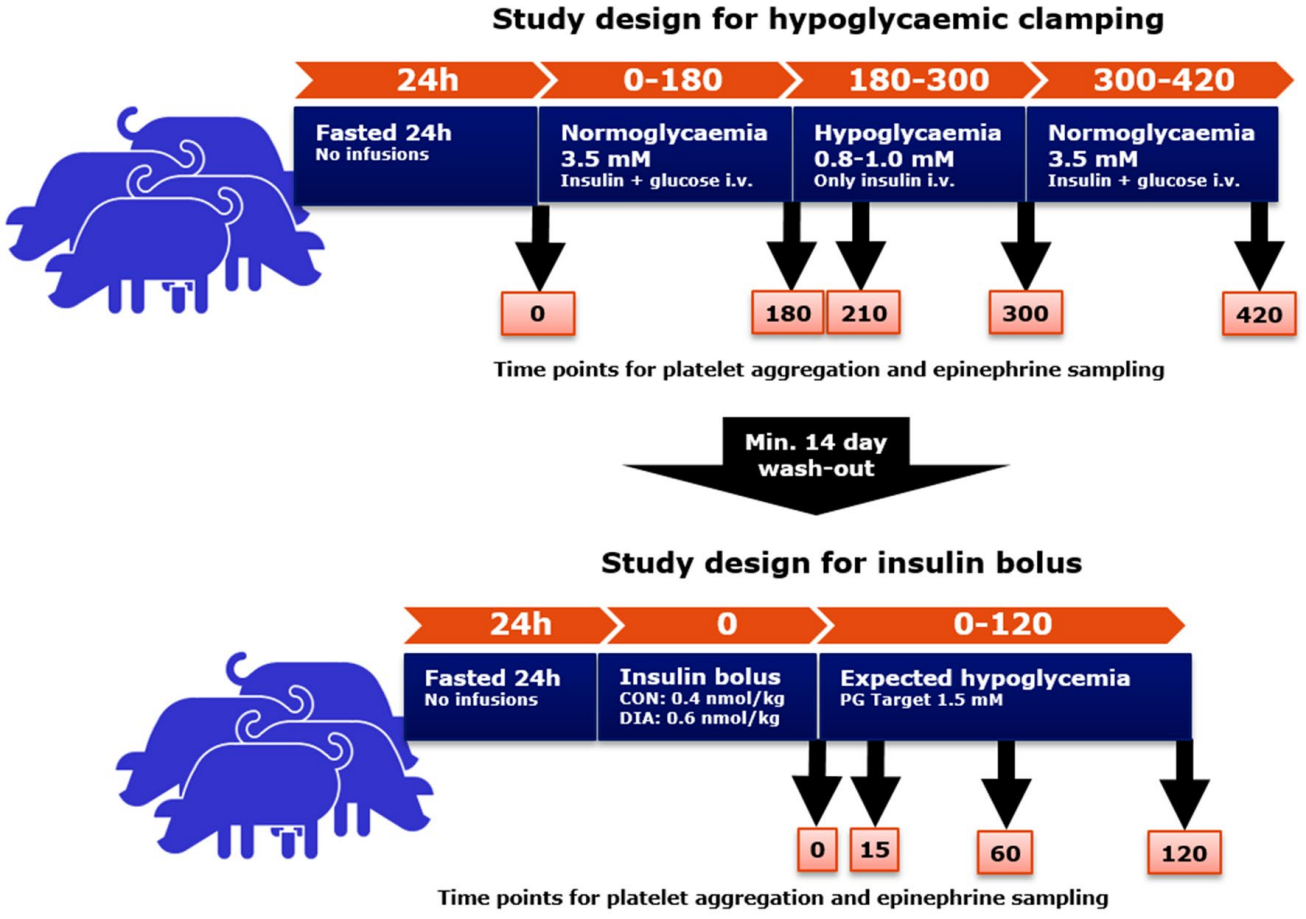
Study design. Hyperinsulinaemic, hypoglycaemic clamp protocol: Pigs were fasted for 24 h prior to study. Pigs received a constant infusion of human insulin (16 pmol/kg/min) throughout the study. A variable glucose infusion (GIR) was given to regulated plasma glucose (PG) to a desired level. PG was measured every five to fifteen minutes. PG target for the first 180 min of study was 3.5 mM (normoglycaemia). After 180 min, GIR was completely turned off, without turning of the insulin, to induce a rapid drop in PG. After initial drop, GIR were carefully increased on to obtain PG target of 0.8–1.0 mM (hypoglycaemia). At 300 min, GIR was increased to obtain normoglycaemia until study end at 420 min. Time-points for epinephrine and platelet aggregometry sampling is illustrated below. Insulin bolus protocol: the bolus protocol was initiated following a minimum 14 day wash-out period. Pigs were fasted for 24 h prior to study. At time 0 min, pigs received an intravenous human insulin bolus (control group (CON) 0.4 nmol/kg, diabetic (DIA) group 0.6 nmol/kg). PG was measured every five to fifteen minutes. Time-points for epinephrine and platelet aggregometry sampling is illustrated below. Software used to generate graphics: Microsoft PowerPoint 2016.

For both studies, blood samples were drawn from a vena cava catheter at predetermined time-points (Fig. 1).

### Clinical signs of hypoglycaemia

During both protocols, clinical signs of hypoglycaemia (ataxia, apparent nausea with or without vomiting, mild myoclonic activity or “tics”, severe myoclonic activity and signs of fatigue) were scored (0 or 1).

### Circulating biomarkers

Glucose was measured from EDTA plasma continuously during both studies (YSI-2900, YSI Inc. OH, USA). For plasma glucagon, human insulin and C-peptide analysis, whole blood was added to 30 µL aprotinin (Trasylol 10.000 KIE/mL, Nordic Drugs AB, Limhamn, Sweden) in a 1.5 mL EDTA coated tube to prevent glucagon degradation. Glucagon, C-peptide and human insulin was quantified using in-house developed luminescence oxygen channelling immunoassays. Lower limit of quantification (LLOQ) for glucagon, C-peptide and human insulin was 4, 45 and 10 pM, respectively.

For plasma epinephrine analysis, whole blood was added to ice-cold EGTA (2.88 mg/mL) and glutathione (1.44 mg/mL) solution (E3889 and G4251, Merck Life Science A/S, Søborg, Denmark) and centrifuged at 3000G for 10 min at 4 °C within 30 min of sampling. Plasma was stored at -80 °C and analysed within 6 months. Epinephrine levels was quantified using liquid chromatography-mass spectrometry with a LLOQ of 0.05 ng/mL as previously described^25^.

Serum biochemistry was analysed using the Advia 1800 Chemistry System (Siemens Healthcare A/S, Ballerup, Denmark). Acute phase proteins C-reactive protein and serum amyloid A was determined as described elsewhere^26^. Haematology parameters was determined in fresh EDTA-stabilized whole blood (Sysmex XT-2000iv, Sysmex Nordic ApS, Copenhagen, Denmark).

### Continuous electrocardiography (ECG)

Continuous ECG was recorded with a Lifecard CF recorder (Spacelabs Healthcare, WA, USA). Electrodes were placed as previously described: “Lead I” (neck (A)—processus xiphoideus (B) and “lead II” (manubrium sterni (C)—processus xiphoideus (D))^27^. All pigs were dosed with 8–10 mL propofol i.v. (Propofol “B. Braun” 10 mg/mL, B. Braun Medical A/S, Frederiksberg, Denmark) two hours prior to study start to ensure stress-free handling when placing equipment.

ECG recordings were analysed using Pathfinder SL (Spacelabs Healthcare, WA, USA). The recording was manually edited by adjustment of R-wave indicators and arrhythmia detection, if needed. Observer was blinded to pig identification, group and time setting. Number of arrhythmic events (supraventricular ectopic beats, ventricular extra-systole and 2^nd^ degree atrioventricular blocks^28^), ST-segment derivations, QT-interval duration and T-wave morphology were registered during normoglycaemia, hypoglycaemia and normoglycaemia after hypoglycaemia. T-wave amplitude (measured from the isoelectric line of TP segment, to highest point of deflection^29^), ST-segment derivation (distance from PQ segment to J-point^30^) as well as QT-interval duration (corrected for heart rate using Bazett’s formula^29^) were measured as a mean of ten consecutive sinus beats using ImageJ (National Institute of Health Sciences, Kawasaki, Japan).

Frequency-domain HRV was calculated from predetermined, five-minute epochs using the build-in Fast Fourier Transformation of Pathfinder SL. Very low frequency power bands at 0.015–0.05 Hz (VLF), low frequency at 0.05–0.15 Hz (LF), high frequency at 0.15–0.40 Hz (HF) and LF to HF ratio (LF/HF) were reported^31^.

### Light transmission platelet aggregometry

Platelet rich (PRP) and platelet poor plasma (PPP) were prepared at previously described^32^ and 250 µL of PPP and 230 µL of PRP were transferred to glass tubes containing a stirring magnet in an aggregometer (PAP-8E, Bio/Data Corporation, PA, USA). PPP represented a light signal of 100% aggregation. 20µL of ADP (HB-5502-FG, Hart Biologicals, Hartlepool, UK) diluted in modified Hepes-Tyrodes buffer was added to PRP. Each pig’s PRP was stimulated with 0, 1, 2, 4, 6, 8, 10 and 20 µM ADP to generate dose–response curves. The light transmission was recorded for 10 min and was measured every 0.1 min. Maximum aggregation response (MAX) and area under the curve (AUC) were calculated for each dose of ADP. In addition, effective concentration of ADP to generate 50% response was calculated for both MAX (EC50_MAX_) and AUC (EC50_AUC_).

### Statistical analyses

Linear mixed effect models with individual pig as random variable were used to analyse endocrine, ECG and platelet aggregation responses during hypoglycaemia using SAS 9.4 (SAS Institute, Cary, USA). Group and time were used as explanatory variables, and interaction between group and time was included as well. The models were reduced by backward elimination. Logarithmic transformation was used to enhance the fit of the models if needed. Differences between groups in baseline values of circulating biomarkers, total insulin exposures and basal characteristics (Mann–Whitney U test), and presence of clinical signs of hypoglycaemia (Fisher’s Exact test) were tested. Graphical representation and calculations of EC50 were made using GraphPad Prism 8 (GraphPad Software Inc., San Diego, USA). The level of significance was set at *P* < 0.05. Data is reported as median and interquartile range.

### Conference presentation

Part of this work has been presented as a poster at the 56th annual meeting of the European Association for the Study of Diabetes.

## Results

The experimental procedures were generally well tolerated by the animals, however, three control pigs were excluded due to dysfunctional catheters (n = 2) or ECG abnormalities (n = 1) before study start. One diabetic pig was included in the BOLUS protocol, but was euthanized before the CLAMP due to clinical signs of systemic infection. Basal characteristics of the pigs are presented in Table 1.

**Table 1:** Basal data.

	Control		Diabetic		*P*-value	
	Clamp	Bolus	Clamp	Bolus	Clamp	Bolus
Age (months)	11.8 (11.7–12.2)		16.7 (15.3–17.5)		0.004	
Weight (kg)	25.0 (23.9–26.1)	30.0 (30–31.5)	36.6 (35.0–38.3)	36.0 (34.8–37.2)	0.004	0.004
Daily insulin glargine (IU)	–	–	26 (23–28)	25 (18.5–26.3)	–	–
CRP* (µg/mL)	51.1 (32.9–78.3)	45.1 (33.3–134.5)	33.9 (27.6–44.9)	39.4 (19.3–60.3)	ns	ns
SAA† (µg/mL)	17.4 (3.9–37.5)	3.9 (3.9–104.2)	55.6 (16.6–148.2)	12.75 (3.9–30.8)	ns	ns
Albumin (g/L)	43.85 (43.33–45.15)	45.89 (42.13–49.92)	61.67 (47.82–73.14)	53.76 (50.20–61.33)	ns	0.030
D3 hydroxybutyrate (mM)	0 (0–0.01)	0.01 (0.01–0.02)	0.03 (0.02–0.05)	0.05 (0.02–0.09)	0.042	0.033
Potassium (mM)	4.3 (4.1–4.4)	4.4 (4.1–4.4)	5.4 (4.4–6.6)	4.7 (4.4–4.8)	ns	0.028
Creatinine (µM)	73 (65–76)	74 (65–83)	94 (79–111)	80 (73–87)	ns	ns
Urea nitrogen (mM)	2.6 (2.2–3.0)	2.5 (2.3–3.2)	3.6 (2.6–4.8)	2.7 (2.4–3.6)	ns	ns
Hematocrit (%)	33.0 (28.7–33.5)	30.1 (27.9–33.5)	32.4 (30.3–36.1)	35.7 (31.2–38.1)	ns	ns
Red blood cells (10^12^/L)	6.6 (5.9–7.0)	6.2 (5.6–6.6)	5.2 (4.6–6.2)	6.2 (5.1–6.6)	ns	ns
White blood cells (10^9^/L)	13.7 (9.0–15.0)	11.9 (9.7–14.8)	7.3 (6.1–10.6)	6.9 (5.8–7.4)	0.03	0.004
Platelets (10^9^/L)	488 (400–595)	512 (364–610)	358 (261–606)	398 (363–480)	ns	ns

Selected baseline data obtained from both groups obtained at the start of studies, including age, weight, serum biomarkers and complete blood count. Data shown as medians and interquartile range. Significant difference between groups set to *P* < .05 and tested by Mann–Whitney U-test. *CRP = C-reactive protein, †SAA = serum amyloid A.

### Counter-regulatory endocrine response to hypoglycaemia

**Figure 2:**
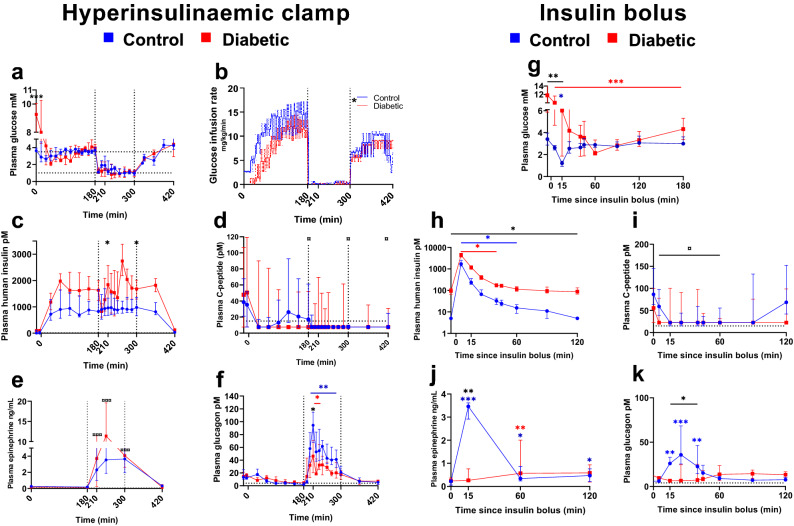
Endocrine response. Plasma glucose mM (**a** + **g**), human insulin pM (**c** + **h**), plasma C-peptide pM (**d** + **i**), plasma epinephrine ng/mL (**e** + **j**) and plasma glucagon pM (**f** + **k**), measured during the hyperinsulinaemic clamp and insulin bolus study. Glucose infusion rate during clamp in mg/kg/min (**b**). Clamp: blue lines: control pigs, red lines: diabetic pigs. Bolus: green lines: control pigs, orange lines: diabetic pigs. Data shown as medians ± interquartile range, except in glucose infusion rate, where each individual is illustrated. Significant difference to baseline within group is illustrated with accordingly colored stars (*), and, when both groups are considered together, as black currency sign (**¤)**. Significance between groups as black stars (*). *P < .05, **P < .001, ***P < .0001, by linear mixed modelling or Mann–Whitney U-test. Horizontal dotted lines: lower limit of quantification of assay. Vertical lines: start and end of hypoglycaemia in the clamp. Software used to generate graphs: GraphPad Prism 8 (GraphPad Software Inc., San Diego, USA).

It was possible to induce hypoglycaemia in both CON and DIA using the CLAMP protocol to target level of 0.8–1 mM (Fig. 2a). In the CLAMP, total insulin exposure did not differ between groups (CON 891 pM (721–1319), DIA 1630 pM (1235–2110)), however, temporal differences were seen between groups (see Fig. 2c). In the BOLUS, control pigs received 0.4 nmol/kg and diabetic pigs 0.6 nmol/kg human insulin resulting in total exposure of 12.5 nM (9.0–19.0) and 74.1 nM (65.2–88.8), respectively (*P* = 0.004) (Fig. 2h). Total glucose infusion during CLAMP was higher in CON (3130 mg/kg (2508–3332)) compared to DIA (2178 mg/kg (1874–2542)) (*P* = 0.02), indicating a higher insulin sensitivity in CON (Fig. 2b).

Baseline plasma C-peptide levels did not differ between groups in either the CLAMP (Fig. 2d) or BOLUS (Fig. 2i). In the CLAMP, C-peptide decreased from start of hypoglycaemia until end of study for both groups and likewise, in the BOLUS, from t15 to t60 (Fig. 2d,i), indicating suppression of endogenous insulin production.

At baseline in the CLAMP protocol, before infusions, DIA had significant higher PG levels compared to CON (Fig. 2a). No difference in median PG appeared during the first three hours of normoglycaemia (CON: 3.52 mM (3.29–3.60); DIA: 3.42 mM (2.94–4.23), and, during two hours of hypoglycaemia (CON: 1.30 mM (1.09–1.66); DIA: 1.16 mM (1.01–1.32)).

The epinephrine response to hypoglycaemia in CLAMP did not differ significantly between CON and DIA. When considering both groups together, plasma epinephrine levels increased approximately 25-fold after one hour of hypoglycaemia (5.13 ng/mL (3.19–11.60)) compared to baseline (0.2 ng/ml (0.18–0.30)) (Fig. 2e) with a return to baseline level at end of study.

In the bolus protocol, only CON reached hypoglycaemic target with PG nadir at 1.21 mM (0.89–1.46) after 15 min. PG nadir for DIA was 2.14 mM (1.87–4.02) after 60 min (Fig. 2g).

In the BOLUS, CON had approximately 15-fold increase in epinephrine at fifteen minutes compared to baseline. DIA had increased epinephrine compared to baseline only at 60 min post insulin dose (Fig. 2j).

Plasma glucagon increased approximately six-fold for CON (89.8 pM (67.7–118.5)) and three-fold for DIA (46.3 pM (20.5–70.9)) compared to baseline (CON: 14.9 pM (9.6–25.3), DIA: 14.8 (14.4–21.2)) in both groups during the CLAMP, with highest levels after 30 min (t210) of hypoglycaemia, with significantly higher levels in CON compared to DIA (see Fig. 2f). In the BOLUS, only CON had an increase in glucagon in response to insulin bolus (see Fig. 2k).

### Clinical signs of hypoglycaemia

All pigs displayed clinical signs of fatigue during CLAMP (*P* < 0.0001), and during BOLUS it was observed all pigs in CON and 4/6 in DIA (*P* = 0.0002). During CLAMP, ataxia (1/5 CON, 3/5 DIA), mild myoclonic tics (1/5 CON, 2/5 DIA), severe myoclonic activity (1/5 DIA) and vomiting (1/5 DIA) were observed. No other signs than fatigue was seen during BOLUS.

### Electrocardiographic changes

**Figure 3:**
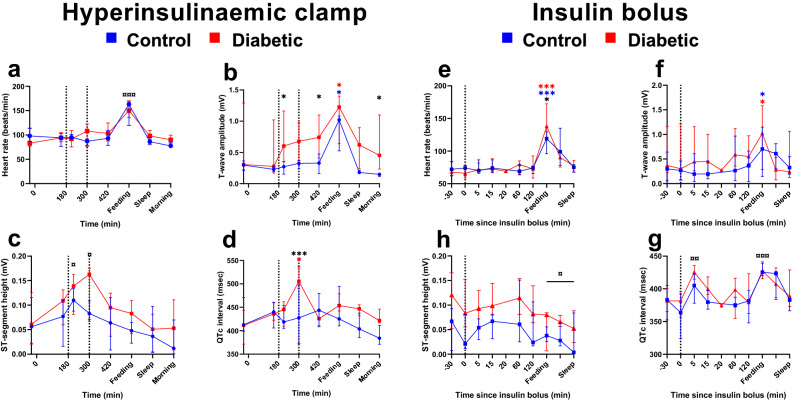
Electrocardiographic morphology. Measures of changing electrocardiographic morphology. Heart rate in beats-per-minute (**a** + **e**), amplitude of the T-wave in mV (**b** + **f**), height of the ST-segment in mV (**c** + **g**) and heart rate corrected duration of the QT-interval (**d** + **h**) during hyperinsulinaemic clamp and insulin bolus study. Clamp: blue lines: control pigs, red lines: diabetic pigs. Bolus: green lines: control pigs, orange lines: diabetic pigs. Data as medians ± interquartile range. Significant difference to baseline within group is illustrated with accordingly colored stars (*), and, when both groups are considered together, as black currency sign (**¤)**. Significance between groups as black stars (*). *P < .05, **P < .001, ***P < .0001, by linear mixed modelling. Vertical lines: start and end of hypoglycaemia in the clamp or insulin bolus time. Software used to generate graphs: GraphPad Prism 8 (GraphPad Software Inc., San Diego, USA).

Heart rate was not influenced by hypoglycaemia (Fig. 3a,e). In the CLAMP, QTc-interval was prolonged in DIA after two hours of hypoglycaemia (*P* < 0.0001), independently of heart rate, while CON that had no changes in QTc (Fig. 3d). In the BOLUS, QTc did not differ between CON and DIA, but QTc increased five minutes after insulin bolus and at feeding (Fig. 3h, P < 0.001), with the increase during feeding most likely accounting to an over-correction due to high heart rate. During hypoglycaemia in the CLAMP, T-wave amplitude was higher in DIA compared to CON (Fig. 3b) while no changes were seen in BOLUS (Fig. 3f). The ST-segment was elevated from baseline, with no difference between groups (Fig. 3c). No increase in ST elevation was seen during hypoglycaemia in the bolus protocol, even though target PG (< 1.5 mM) was reached in CON. A decrease in ST-segment elevation compared to baseline was seen at feeding and sleep after the study, with no difference between groups (Fig. 3g).

In both CON and DIA, total number of arrhythmias increased significantly in CLAMP during hypoglycaemia to 2.25 events/hour compared to the initial normoglycaemia period of 0.04 events/hour (*P* = 0.03) with no difference between groups. Number of ventricular ectopic beats changed from 0 (0–0) and 0.17 (0–0.33) during normoglycaemia to 0 (0–0.5) and 1.0 (0–1.5) per hour during hypoglycaemia in CON and DIA respectively. Number of supraventricular premature beats changed from 0 (0–26) and 0 (0–5.67) to 0.5 (0–39) and 0 (0–25) per hour for CON and DIA respectively. Second degree atrioventricular blocks changed from 0 (0–0) and 0 (0–0) to 0 (0–8.5) and 0 (0–0) per hour in CON and DIA respectively. In BOLUS, hypoglycaemia and group did not influence in total number of arrhythmic beats.

### Heart rate variability

**Figure 4:**
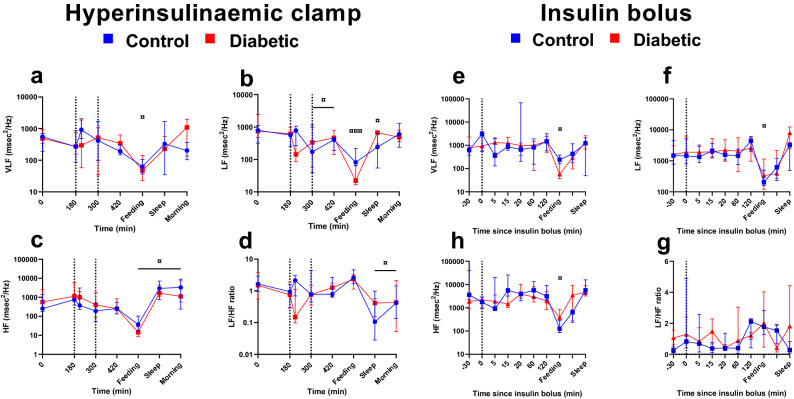
Frequency domain heart rate variability. Very low frequency band power (VLF) in msec^2^/Hz (**a** + **e**), low frequency band power (LF) in msec^2^/Hz (**b** + **f**), high frequency band power (HF) in msec^2^/Hz (**c** + **g**) and ratio between LF and HF power (**d** + **h**) during hyperinsulinaemic clamp and insulin bolus study. Clamp: blue lines: control pigs, red lines: diabetic pigs. Bolus: green lines: control pigs, orange lines: diabetic pigs. Y-axis is logarithmic. Data as medians ± interquartile range. Significant difference between groups as black stars (*). Significant difference to baseline within group is illustrated with accordingly colored stars (*), and, when both groups are considered together, as black currency sign (**¤)**. *P < .05, **P < .001, ***P < .0001, by linear mixed modelling. Vertical lines: start and end of hypoglycaemia in the clamp or insulin bolus time. Software used to generate graphs: GraphPad Prism 8 (GraphPad Software Inc., San Diego, USA).

During hypoglycaemia in the CLAMP, only the LF frequency band power decreased (*P* = 0.004) with no difference between groups (Fig. 4a–d). In the BOLUS, hypoglycaemia did not influence HRV (Fig. 4e–h). The VLF, LF, HF frequency band power and LF/HF ratio decreased during feeding after both protocols. After the CLAMP, the LF and HF bands increased during night and in the morning after study (*P* < 0.05) while the LF/HF ratio decreased accordingly at these time points as well (*P* < 0.05) (Fig. 4).

### Light transmission platelet aggregometry

**Figure 5:**
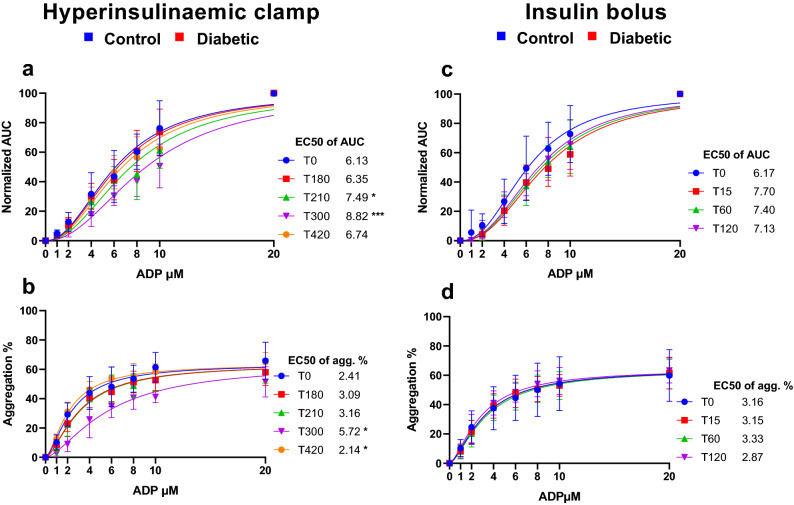
Platelet aggregometry. Light transmission platelet aggregometry, with no distinction between groups, at different time-points throughout the studies. Normalized (0–100%) area under the curve (AUC) at different concentrations of adenosine diphosphate (ADP) (**a** + **c**), and maximum aggregation percentage at different concentrations of ADP (**b** + **d**), both with overlays of fitted dose–response curves at the different time points. Hyperinsulinaemic clamp: blue line: time 0 representing baseline dose–response to ADP, red line: after 180 min representing two hours of normoglycaemia, green line: after 210 min representing 30 min of hypoglycaemia, purple line: after 300 min representing two hours of hypoglycaemia, orange line: 420 min representing two hours of normoglycaemia after hypoglycaemia. Data shown as medians ± interquartile range. Insulin bolus: blue line: time 0 representing baseline dose–response to ADP, red line: 15 min after insulin bolus, green line: 60 min after insulin bolus, purple line: 120 min after insulin bolus. Significant difference to baseline effective concentration for 50% reaction (EC50) labeled as stars (*) set to *P < .05 and ***P < .0001 by linear mixed modelling. Software used to generate graphs: GraphPad Prism 8 (GraphPad Software Inc., San Diego, USA).

The platelet aggregation response decreased during hypoglycaemia in CLAMP. EC50_AUC_ increased, reflecting decreased platelet aggregation response, at both 30 (7.73 (6.45–8.51), *P* = 0.03) and 120 min (9.19 (7.42–9.80), *P* < 0.0001) of hypoglycaemia compared to baseline (6.58 (6.18–6.98)) with no difference between groups (Fig. 5a). Furthermore, EC50_MAX_ was increased after 120 min of hypoglycaemia (baseline; 3.24 (2.55–5.39), t300; 6.11 (4.32–6.86), *P* < 0.01), but slightly decreased at the end of the clamp (t420 2.52 (2.17–3.11), *P* < 0.01) (Fig. 5b).

Platelet aggregation response was not influenced by hypoglycaemia during the BOLUS protocol (Fig. 5c,d).

## Discussion

In the present study it was possible to induce hypoglycaemia close to target of 1.0 mM plasma glucose using the clamp approach in both healthy and diabetic Göttingen minipigs. Using the bolus approach, only healthy pigs reached hypoglycaemic target while the diabetic group showed a variable response to the insulin bolus, reflecting variation in initial fasted plasma glucose and insulin sensitivity among animals in this STZ induced diabetes model. Thus, to use the bolus setup for induction of insulin-induced hypoglycaemia in STZ diabetic minipigs may require an initial insulin sensitivity determination in the individual animals. The healthy minipigs used in this study had a lower fasting blood glucose than what has been reported in studies using commercial pig breeds^33^, rats^15^ and in healthy humans^34^, prompting a need for a quite low plasma glucose target to achieve hypoglycaemia with clinical signs. In this study, all minipigs displayed signs of fatigue during hypoglycaemia in the clamp setup, and some even signs of neuroglycopenia, such as myoclonic activity and ataxia^35^, some of which has been described in rat studies^14, 15^ but not in a previous study of hyperinsulinaemic hypoglycaemia in awake pigs^33^. Though the clamp approach seems to be the better option to reliably induce hypoglycaemia to a specific target in both healthy and diabetic pigs, the single insulin bolus might reflect the clinical setting more closely, where patient’s insulin requirements and insulin delivery might not always match^35^.

The counter-regulatory autonomous response to hypoglycaemia in the clamp protocol induced an approximately 25-fold increase in plasma epinephrine, an increase similar to what has been found in human studies, where autonomous symptoms of hypoglycaemia were also recorded^8^. Release of epinephrine is a counter-regulatory mechanism to hypoglycaemia observed in humans^22^ and in rodent models^14, 15^, but in healthy pigs, no response or only a weak response have been reported^33, 36^. In the bolus setup, only the healthy group reached hypoglycaemic target to induce an epinephrine response and the lack of response in DIA is likely driven by the low insulin sensitivity, where even a 50% higher insulin dose than given the healthy group was insufficient to induce hypoglycaemia at target level.

Electrocardiography by wearable devices has been used in human studies to investigate connections between glucose fluctuations and ECG changes^37^. Cardiac arrhythmias during hypoglycaemia have been reported in both human^10, 13^ and rat studies^14, 15^, suggesting arrhythmias as potential triggers for dead-in-bed syndrome. In the present study, the most commonly observed type of arrhythmic beats were supraventricular ectopic beats, with the overall rate of arrhythmias increasing during hypoglycaemia, possibly reflecting the proarrhythmic effect of hypoglycaemia. In a clinical study of spontaneous hypoglycaemia, supraventricular ectopic beats are the most common arrhythmia presenting^12^ while another study report increase in both ventricular and supraventricular ectopic beats^11^. Hypoglycaemia also induces change in ventricular repolarization, causing QT-interval prolongation but also altered T- and U-wave morphology in humans^38^. In this study, diabetic pigs had a higher amplitude of T-waves during and after hypoglycaemia compared to healthy controls, a change contrary to what is seen in humans during hyperinsulinaemic hypoglycaemia, where hypokalemia also often is present^8, 38, 39^. Prolongation of the QT-interval during hypoglycaemia is thought to be an effect of sympathetic stimulation and hypokalemia^39^, a pro-arrhythmogenic condition causing reduced repolarization of cardiomyocytes. The effects of hypokalemia is most often expressed in prolonged QT-interval, flattening of T-waves and can cause afterdepolarization-mediated arrhythmias^40^, and the arrhythmogenic effect of hypokalemia can be somewhat reversed by potassium intravenous infusion in a rodent model of hyperinsulinaemic hypoglycaemia^15^. Importantly, in the present study, significantly increased QTc-interval without change in heart rate was seen during hypoglycaemia, which corresponds to what has previously been reported in hypoglycaemia in humans and rats^9, 15^. Interestingly, in our bolus study, QTc-interval increased only five minutes after insulin dosing, and before reaching hypoglycaemia target. This suggests QTc-interval as a potential early marker of insulin-induced hypoglycaemia that need to be investigated further. A slight ST-segment elevation was also seen during hypoglycaemia, which might indicate myocardial hypoxia, a phenomenon that has been reported sporadically in people with type 1 diabetes and cardiovascular disease^20^. The finding of relative few ECG changes during hypoglycaemia in the present study can due to the hypoglycaemic level chosen in this study was not severe enough to induce the changes observed in humans, as has already been demonstrated in a rat study with hypoglycaemic target of 0.55–0.83 mM plasma glucose^15^. However, it is also possible, that the same degree of changes observed in rats and human patients cannot be induced in the pig due to species differences.

Frequency domain analysis of heart rate variability has been investigated in people with diabetes, and changes in frequency band power has been associated with altered autonomic nervous control of the heart and fluctuations in blood glucose levels^37^. In the present study, only the LF band power decreased during hypoglycaemia in groups, with no other frequency bands changing. This decrease in the LF band has been demonstrated in experimental human studies of hypoglycaemia corresponding to an increase in plasma epinephrine^41^ or in hypoglycaemia detected by CGM in type 1 diabetic patients^12^. Conversely, the LF band is thought to reflect baroreceptor activity in resting states, which can be influenced primarily by parasympathetic stimulation, but also blood pressure and sympathetic stimulation^42^, suggesting a counter-regulative parasympathetic cardiac modulation to elevated epinephrine during hypoglycaemia. Interestingly, parasympathetic blockade has been associated with increased survival in severe hypoglycaemia in rats^14^. The only other change seen in all frequency bands was during feeding, which is to be considered a positive control of sympathetic simulation during excitation^31^.

In the present study, hypoglycaemia induced glucagon release, but diabetic pigs had an attenuated glucagon response to hypoglycaemia in the clamp compared to healthy pigs, comparable to the human counter-regulatory response in people with and without type 1 diabetes^43^. Furthermore, it should be noted that the alpha-cells can also be damaged during the STZ treatment to induce diabetes, which may explain the lower glucagon release^44^. Both groups had a decrease in C-peptide during clamp and bolus, reflecting the decrease in endogenous insulin secretion during hypoglycaemia due to suppression by the exogenous insulin. Diabetic pigs had a higher level of human insulin in plasma, even though they received the same dosage as the healthy pigs, as well as a lower glucose infusion rate to maintain target plasma glucose, further supporting the observation of a lower insulin sensitivity in this group.

Platelets are important players in the cardiovascular pathophysiology underlying the formation of atherosclerosis and coronary heart disease^3^. The most common comorbidity of diabetes is cardiovascular disease, and people with diabetes have increased platelet activation and are often resistant to anti-platelet therapy^45^. Platelet hyperreactivity has also been demonstrated in the Ossabaw pig model of metabolic syndrome^46^ and in pigs with alloxan-induced diabetes^47^. However, in the present study, no difference between healthy and diabetic pigs in platelet activation was observed, indicating that the diabetic minipig model employed here does not reflect this aspect of the cardiovascular pathophysiology associated with diabetes, at least not during insulin treatment.

Healthy humans subjected to an insulin bolus to induce hypoglycaemia has been demonstrated to have increased platelet aggregation, which persists after hypoglycaemia is resolved^21^. No such effect was found in our bolus study, and in the clamp study, the opposite effect was seen, with hypoglycaemia decreasing the platelet aggregation responses. Interestingly, previous studies of healthy humans exposed to a hyperinsulinaemic euglycemic clamp demonstrated inhibition of platelet reactivity^48^ similarly to what we observe in the present study, causing the speculation that the reduced platelet reactivity may a result of high insulin exposure, since insulin has been demonstrated to exert a inhibitory effect on platelet reactivity in human platelets^48^.

The present study is limited by the low number of animals included and differences in age and weight between the diabetic and healthy group. The differences in age and weight between groups were due to the diabetic pigs were included in other studies prior to this study. Though the diabetic group weighted more than the control group, both groups were close to the normal weight for their age^49^. The pigs in the present study had elevated inflammatory markers compared to previous porcine studies^50^, indicating some sort of subclinical inflammatory state, most likely due to the permanent central venous catheters. Diabetic pigs had slightly elevated baseline serum potassium compared to the healthy pigs, possibly creating a much steeper slope of possible hypokalemia, a common effect of hyperinsulinaemic hypoglycaemia, which can lead to ECG changes^38^. Propofol was used as a brief sedation agent to apply ECG monitors to the pigs prior to the start of study. Though propofol is known to have a short sedative duration, it cannot be excluded, that the dosing of propofol might alter the counter-regulatory response to hypoglycaemia. A limitation of this study is also the lower fasting blood glucose in the healthy Göttingen minipig compared to humans, making a direct translation of glycaemic levels more difficult. In the rat, fasting blood glucose has been shown to be 5.8–7.1 mM, which matches the human fasting level more closely^15^. Moreover, continuous glucose monitoring was not included in the diabetic group. Plasma glucose was only measured in the morning, and thereby it cannot be excluded, that the pigs had experienced hypoglycaemia prior to the study. However preceding hypoglycaemic episodes were not expected due to high fasted morning plasma glucose levels in the diabetic group.

Overall, the novel Göttingen minipig hyperinsulinaemic-hypoglycaemic clamp model appear to be a highly clinically relevant translational model. The counter-regulatory endocrine response to hypoglycaemia seems to closely match that of humans, with clinically significant glucagon and adrenalin increase, making it possible to use minipigs as models for possible intervention studies of hypoglycaemia. The electrocardiographic changes and heart rate variability fluctuations observed also match what has previously been observed in humans, which further corroborates the relevance of the model. However, the electrocardiographic changes were mainly mild and transient, prompting a need for studies conducted in more severe hypoglycaemic states to better understand the minipig as a possible model of dead-in-bed syndrome and the proarrhythmic effects of severe hypoglycaemia, which for ethical reasons cannot be studied experimentally in humans. Furthermore, the effect of recurrent hypoglycaemia needs to be examined to determine if the physiological effects are comparable to human studies^51^.

## Data Availability

The datasets generated during and/or analysed during the current study are available from the corresponding author on reasonable request.
